# Screening of Concentration and Antimicrobial Effectiveness of Antimicrobial Preservative in Betastatin Besylate Nasal Spray

**DOI:** 10.1155/2020/1315069

**Published:** 2020-12-12

**Authors:** Yanhong Sun, Yinhuan Wang, Jianming Zhou, Qingxue Zhou, Shilei Dong

**Affiliations:** ^1^Department of Clinical Laboratory, The Children's Hospital, Zhejiang University School of Medicine, National Clinical Research Center for Child Health, Hangzhou 310052, China; ^2^NMPA Key Laboratory for Testing and Warning of Pharmaceutical Microbiology, Zhejiang Institute for Food and Drug Control, Hangzhou 310052, China; ^3^Department of Clinical Laboratory, Hangzhou Maternity Hospital, Hangzhou 310008, China; ^4^Department of Clinical Laboratory, Zhejiang Hospital, Hangzhou 310013, China

## Abstract

**Objective:**

To explore the optimal concentration and antimicrobial effectiveness of antimicrobial preservative in betastatin besylate nasal spray.

**Methods:**

By using *Staphylococcus aureus*, *Pseudomonas aeruginosa*, *Escherichia coli*, *Candida albicans*, and *Aspergillus niger* as test strains, the antimicrobial effectiveness of betastatin besylate nasal spray containing different concentrations of antimicrobial preservative (0.02%, 0.0125%, and 0.005% benzalkonium chloride, respectively) was determined by using bacteriostatic effect test (Chinese Pharmacopoeia, 2015 edition).

**Results:**

The antimicrobial effectiveness of betastatin besylate nasal spray containing 0.02% and 0.0125% benzalkonium chloride, respectively, complied with the regulations of Chinese Pharmacopoeia (2015 Edition) against five test strains. However, the antimicrobial effectiveness of betastatin besylate nasal spray containing 0.005% benzalkonium chloride against *P. aeruginosa* did not meet the requirements of Chinese Pharmacopoeia.

**Conclusion:**

Benzalkonium chloride at a concentration of 0.125% can be used as an added antimicrobial preservative in betastatin besylate nasal spray.

## 1. Introduction

Betastatin besylate is a highly selective histamine H1 receptor antagonist, which has a stabilizing effect on mast cells and can effectively inhibit the infiltration of eosinophils into the inflammation site during allergic inflammation. It can also inhibit the activation of eosinophils, interleukin (IL)-5, leukotriene B4 (LTB4), and platelet activating factor, thereby alleviating allergic inflammation. Betastatin besylate is already widely used to improve the symptoms of allergic rhinitis, urticaria, and chronic itching. It has been found that there are betastatin besylate tablets, and tablets are generally packaged in a single dose and do not need to add antimicrobial agents. However, betastatin besylate nasal spray is not available yet. Use of betastatin besylate as a nasal spray, which can be locally administered to the nasal cavity, can enhance the therapeutic effect and rapidly relieve clinical symptoms such as nasal congestion, runny nose, and itchy nose [1–3].

With increasing smog in the atmosphere, allergens causing allergic diseases in children are becoming increasing diversity, especially into the winter season, resulting in an increase in the incidence of allergic rhinitis among children [4]. Betastatin besylate can quickly alleviate allergic symptoms, and thus, the purpose of the present study was to determine the type and concentration of bacteriostatic agents that can be used in betastatin besylate nasal spray for clinical use. As betastatin besylate nasal spray is not packaged as a single dose and does not possess sufficient antimicrobial efficacy, the risk of microbial contamination is extremely high during normal storage and multiple use, which can easily lead to deterioration of the drug and affect the user. Hence, it is necessary to add an appropriate quantity of a bacteriostatic agent to ensure the quality and safety of betastatin besylate nasal spray [5].

Benzalkonium chloride is a quaternary ammonium salt compound, which affects the permeability of bacterial membrane by destroying the interaction between bacterial cell wall lipopolysaccharide and lipid membrane bilayer, leading to leakage and lysis of cytoplasmic components [6]. It is commonly used as an antiseptic and bacteriostatic agent in ophthalmic and nasal drugs [7]. In addition, benzalkonium chloride neither disturbs the nasal blood flow and nasal mucosal temperature in the nasal cavity nor affects the user's therapeutic effect as a preservative [8]. Therefore, to prevent microbial contamination, most of the nasal spray formulations use benzalkonium chloride as an antiseptic [9].

Bacteriostatic efficacy, as an important indicator of microbial evaluation, is a key factor to ensure safety and effectivity of an antimicrobial agent. In principle, an appropriate amount of bacteriostatic agent should meet the requirements of effectiveness, safety, and stability. However, all bacteriostatic agents must ensure safety for use as a medication. The Chinese Pharmacopoeia (2015 edition) General Regulations 1121 bacteriostatic efficacy test clearly stipulates that the amount of bacteriostatic agent used in a medical preparation should be the minimum effective amount and that the effective concentration of the bacteriostatic agent in the final product should be lower than the concentration that is harmful to humans [5]. If the concentration is too low, it may not achieve bacteriostatic effect. On the contrary, if the concentration is too high, there may be toxic side effects. However, the Chinese Pharmacopoeia does not specify the type and concentration of bacteriostatic agents.

Benzalkonium chloride is a bacteriostatic agent added to betastatin besylate nasal spray. Excessive use of benzalkonium chloride can cause symptoms such as palpitations, arrhythmia, and skeletal muscle spasm [10]. In the present study, the Chinese Pharmacopoeia 2015 General Rules 1121 antibacterial efficacy test was employed to explore the acceptable and effective concentration of benzalkonium chloride in betastatin besylate nasal spray. The study provides experimental guidance for the manufacturer to determine the type and concentration of bacteriostatic agent that can be used in betastatin besylate nasal spray during the research and development phase.

## 2. Materials and Methods

### 2.1. Strains and Drugs

The strains used in the experiment are all the strains required by the general principles of Chinese Pharmacopoeia (2015 edition and 2020 Edition), it covers fermented Gram-negative bacteria (*Escherichia coli*) and Gram-positive bacteria (S*taphylococcus aureus*), nonfermentative Gram-negative bacteria (*Pseudomonas aeruginosa*), Candida (*Candida albicans*), and mold (*Aspergillus niger*). Five strains are the representative strains stipulated in the Pharmacopoeia, and they are also the most common strains in the environmental pollution. All the strains used in the experiment were from the China Medical Bacterial Collection Management Center (CMCC), which fully meets the requirements of Chinese Pharmacopoeia and will not affect the experimental results. The strains for the preparation of bacterial suspensions were all third generation.

Betastatin besylate nasal spray (Shanghai Modern Pharmaceutical Preparation Engineering Research Center Co., Ltd. China; specification, 100 *μ*L per spray) containing 2.5 mg of betastatin besylate (7.5 mL per bottle) was used in this study. The nasal spray numbers 71401, 71402, and 71403 contained 0.02%, 0.0125%, and 0.005% benzalkonium chloride, respectively.

### 2.2. Media

Trypticase soy agar (TSA) medium (batch no. 3303005), Sabouraud dextrose agar (SDA) medium (batch no. 20160901), trypticase soy meal broth (TSB) (batch no. 3302035), and Sabouraud dextrose broth (SDB) (batch no. 3302126) were purchased from Guangdong Huan Kai Microbiology Technology Co., Ltd., China. The applicability of the media was in compliance with the Chinese Pharmacopoeia 2015 edition. The media were prepared according to the manufacturer's instructions.

### 2.3. Microbial Suspension Preparation

The microbial suspensions were prepared to appropriate concentrations according to the method described in the Chinese Pharmacopoeia 2015 edition Four General Rules 1121 “Antibacterial efficacy test method.”

### 2.4. Method Suitability Study

In order to meet the requirements of Chinese Pharmacopoeia 2015 edition of the four general rules 1105 “microbial limit test for nonsterile products: microbiological counting method” [5], the recovery rate of betastatin besylate nasal spray with high antibacterial content (No. 71401, containing benzalkonium chloride 0.02%) should not be less than 70%.

It is stipulated that all antimicrobial agents have certain toxicity, and the amount of antibacterial agents in the preparation should be the minimum effective amount in Chinese Pharmacopoeia (2015 edition and 2020 Edition) [5]. When selecting the concentration of benzalkonium chloride, enterprise refers to the data such as the Chinese Pharmacopoeia and FDA “database of inactive substances,” combined with the invention patent “Betastatin Besylate nasal spray and the preparation method” which stipulates that the amout of benzalkonium chloride is 0.005%-0.3%. If the concentration is too low, it may not achieve bacteriostatic effect. On the contrary, if the concentration is too high, there may be toxic and side effects. Therefore, in order to find the lowest effective concentration, three concentration gradients will be set up, and the minimum concentration meeting the standards of the Pharmacopoeia will be found out through the verification of bacteriostatic effect. Therefore, in the initial experiment, we gave priority to the three concentrations starting from the lowest concentration (0.020%, 0.0125%, and 0.005% benzalkonium chloride). Of course, if the first exploration was not successful, we would set the concentration gradient again and continue to study the antibacterial efficacy.

### 2.5. Microbial Inoculation

Sterile bottles (5 bottles for each batch) containing betastatin besylate nasal spray with 0.020%, 0.0125% and 0.005% benzalkonium chloride, respectively, were inoculated with 50 *μ*L of *S. aureus*, *P. aeruginosa*, *E. coli*, *C. albicans*, and *A. niger* at a concentration of about 10^5^–10^6^ colony forming units per mL (CFU/mL). Then, the suspension was mixed well and stored at 23°C in the dark.

### 2.6. Quantification of Viable Microbial Cells

The viable microbial cells were quantified according to the product type and Chinese Pharmacopoeia 2015 edition General Rules 1121 “Antibacterial Effectiveness Check Method”. In brief, 1 mL of the spicemen was collected on days 2, 7, 14, and 28, respectively, inoculated onto a TSA plate (for bacteria) and SDA plate (for fungi), and the number of viable cells was determined by counting and verified by the method suitability test. Finally, the survival numbers of each test bacterium at each interval were calculated and converted into log value.

### 2.7. Results Assessment

The criteria for assessing the antimicrobial effectiveness of the nasal preparation are shown in Table 1. The “reduced log value” in the table refers to the difference between the log value of the number of microbial cells measured at each interval and the log value of the number of microbial cells inoculated in 1 mL of the specimen. Standard “A” indicates the antimicrobial efficacy standard that should be achieved. In special cases, if the antimicrobial agent increased the risk of adverse reactions, standard “B” antimicrobial efficacy was used in the test.

## 3. Results and Discussion

### 3.1. Method Suitability Test

In the preliminary test, betastatin besylate nasal spray solution with a high concentration of bacteriostatic agent (No. 71401, 0.02% benzalkonium chloride) was selected, and *S. aureus*, *P. aeruginosa*, *E. coli*, *C. albicans*, and *A. niger* were added, respectively, to perform the applicability tests by plating and counting methods. The results showed that the recovery rates of *C. albicans* and *A. niger* were both >70%, while that of the other three strains were 0%, which did not meet the requirements of the Chinese Pharmacopoeia, suggesting that the test solution should be further processed by an appropriate method. Subsequently, the applicability test of the abovementioned three bacterial strains was conducted by a membrane filtration method, and the recovery rate of each test strain was noted to be <70%. According to the Chinese Pharmacopoeia 2015 General Regulation 1105, if the inhibitory effect of the specimen on microbial growth could not be eliminated by other methods, then the test solution was neutralized, diluted, or membrane-filtered before adding the test microbial suspension for method suitability test [5]. According to previous research, the bacteria selected the way for the method suitability test which is membrane filtration and then added to the test bacterial suspension, while the fungus selected a counting plate method for method suitability test.

As shown in Table 2, the recovery rate of the method suitability test results could be calculated as follows: (number of colonies in the test group − number of colonies in the specimen control group)/number of colonies in the bacterial control group × 100%, which should not be <70% of that indicated in the Chinese Pharmacopoeia. The bacteriostatic efficacy of the product was determined by a membrane filtration method (Chinese Pharmacopoeia 2015 General Rules 1105 membrane filtration method), whereas the antifungal efficacy was ascertained by plating and counting methods (Chinese Pharmacopoeia 2015 General Rules 1105 plate law).

### 3.2. Antimicrobial Effectiveness

The results obtained revealed that 0.02% and 0.0125% benzalkonium chloride achieved antimicrobial effectiveness standard A against bacteria and fungi (Table 3). However, when 0.005% benzalkonium chloride was employed, the log value of *C. albicans* decreased to 1.7 on day 14, which did not meet the antimicrobial efficacy standard A of the Chinese Pharmacopoeia, but corresponded to standard B. Furthermore, 0.005% benzalkonium chloride did not accomplish standard A or B with respect to the growth of *P. aeruginosa* on day 28, suggesting that the antibacterial efficacy of 0.005% benzalkonium chloride did not meet the Chinese Pharmacopoeia regulations.

## 4. Discussion

The antibacterial effect of bacteriostatic agents is often affected by a number of factors, such as the chemical structure and concentration of the bacteriostatic agent, physical and chemical properties of the active components of the drug, form of the preparation, or storage conditions. In addition, the characteristics of the container used in the experiment, such as the material, shape, volume, and sealing method of the container, may also have a significant influence on the bacteriostatic effect, especially when the material of the container is adsorbed or directly affects the pH of the drug [11, 12]. Therefore, the “Chinese Pharmacopoeia” clearly stipulates that if the amount of each packaging container for the drug preparation is sufficient for experimental use and the container is convenient for inoculating the test microbial strains, mixing, and sampling under aseptic condition, then the test microorganisms should generally be directly inoculated into the original packaging container for analysis. In the present study, as the packaging container of betastatin besylate nasal spray was not convenient for accessing the test microbial strains, mixing, and sampling under aseptic condition, the drug was transferred to a sterile container for analysis.

Bacteriostatic potency test is a long-term, dynamic monitoring process of analyzing the survival of microorganisms in the specimen. It is necessary to examine the inhibition or killing of a large numbers of invading microorganisms by the bacteriostatic agent in a short period of time after inoculation of the specimen. It is also crucial to determine whether the total amount of microorganisms in the specimen can still be controlled within a reasonable range after the microorganisms develop tolerance to the surrounding environment or the stability of the bacteriostatic agent changes over time [13]. The 2005 edition of the Chinese Pharmacopoeia stipulated the preservatives used in some dosage forms, the 2010 edition first included the guiding principles of bacteriostatic efficacy test method, and the 2015 edition switched to the mandatory standard bacteriostatic efficacy test method, implying that the requirements for pharmaceutical bacteriostatic agents are becoming increasingly stringent.

Benzalkonium chloride is an internationally recognized broad spectrum, high efficiency, low toxic disinfectant preservative. Previous studies have reported that a benzalkonium chloride preservative has a strong inhibitory effect on Gram-negative bacteria [14] and has wide applications in the fields of medicine, food, cosmetics, etc. [15, 16]. However, although this type of preservative has low toxicity, it can cause allergies owing to excessive use and can even endanger human health in severe cases [17, 18]. Therefore, it is imperative to explore the minimum effective dose of bacteriostatic agents used in drugs.

In the present study, 0.02% and 0.0125% benzalkonium chloride met the antimicrobial efficacy standard A. In contrast, 0.005% benzalkonium chloride decreased the log value of *C. albicans* to 1.7 on day 14 and thus could not meet the antimicrobial efficacy standard A of the Chinese Pharmacopoeia, but could comply with standard B; however, the observation of *P. aeruginosa* growth on day 28 indicated that this dosage failed to meet standard A or B. In vitro, the antibacterial test found that benzalkonium chloride had the weakest inhibitory effect on *E. coli* and *C. albicans*. The reason for the inconsistency between the two is that the results of the antibacterial efficacy in this study is actually the result of the whole product of betastatin besylate nasal spray, including various raw materials, excipients, and antibacterial agents. However, the antibacterial spectrum of benzalkonium chloride alone cannot fully reflect the antibacterial efficacy of the product, and it is necessary to considered the overall antibacterial effect of the product. In this study, five kinds of bacterial suspensions were added to the betastatin besylate nasal spray to simulate the microbial contamination caused by the repeated opening during use. It is the simulated in situ environment. However, the judgment standard of Pharmacopoeia bacteriostatic efficacy is one significant number. When the test result is 1.7, if only one significant digit is kept, it will become 2, and the A criterion is reached; in this way, the inspector changes from satisfying the B standard to A standard when judging *C. albicans*. This contradiction is expected to be resolved in the future revision of the Chinese Pharmacopoeia.

It must be noted that the criteria for determining the antimicrobial efficacy of nasal preparations differ among the Chinese, European, and US Pharmacopoeia. Nevertheless, the criteria of the Chinese Pharmacopoeia nasal preparations and European Pharmacopoeia nonsterile nasal preparations are consistent, which are stricter than the US Pharmacopoeia. The European Pharmacopoeia and US Pharmacopoeia divide nasal preparations into two categories: sterile and nonsterile nasal preparations. While the diagnostic criteria for antimicrobial efficacy of sterile nasal preparations are more stringent, the Chinese Pharmacopoeia only considers the type and quantity of microorganisms for nasal preparations, and the criteria for determining the antimicrobial efficacy are not differentiated as sterile and nonsterile. Although it is more reasonable to divide the nasal preparation into sterile and nonsterile, the criteria for determining the antimicrobial efficacy of sterile nasal preparation are complex, necessitating higher concentration of the antimicrobial agent to meet the requirements; however, very high inhibitor concentration may cause significant toxicity, thus requiring further research on this standpoint.

## 5. Conclusions

In the current study, it is showed that benzalkonium chloride at a concentration of 0.125% can be used as an added antimicrobial preservative in betastatin besylate nasal spray.

## Figures and Tables

**Table 1:** Assessment criteria for antimicrobial effectiveness of nasal preparation.

		Reduced log value			
		2 d	7 d	14 d	28 d
Bacterial	A	2	3	—	NI
	B	—	—	3	NI

Fungus	A	—	—	2	NI
	B	—	—	1	NI

NI: no increase, indicating that the amount of test strain increased by no more than 0.5 log from the previous measurement time.

**Table 2:** Results of method applicability test.

Test strains	Nasal spray no.	Bacterial control group (CFU)	Test specimen control group (CFU)	Test group (CFU)	Recovery rate (%)	The average recovery rate (%)
*S. aureus*	71401	95 ± 4.2	0	75	78.9	83.9 ± 5.3
	71402		0	79	83.2	
	71403		0	85	89.5	

*P. aeruginosa*	71401	81 ± 2.8	0	70	86.4	90.5 ± 4.4
	71402		0	73	90.1	
	71403		0	77	95.1	

*E. coli*	71401	90 ± 5.7	0	73	81.1	84.1 ± 3.4
	71402		0	75	83.3	
	71403		0	79	87.8	

*C. albicans*	71401	93 ± 8.5	0	70, 66	73.1 ± 3.0	82.1 ± 8.2
	71402		0	80, 76	83.9 ± 3.0	
	71403		0	80, 86	89.2 ± 4.6	

*A. niger*	71401	46 ± 7.1	0	39, 35	80.4 ± 6.2	85.5 ± 4.5
	71402		0	39, 41	87.0 ± 3.1	
	71403		0	42, 40	89.1 ± 3.1	

**Table 3:** Results of antimicrobial effectiveness.

Nasal spray no.	Bacteriostatic concentration	Test strains	Count of bacteria in the specimen (CFU/mL)					Reduced log value						
			Initial value	2 d	7 d	14 d	28 d	Initial value	2 d	7 d	14 d	28 d	28 d compared with 7 d	28 d compared with 14 d
71401	0.02% benzalkonium chloride	*S. aureus*	5.4 × 10^5^	<1	<1	<1	<1	6.3	6.3	6.3	6.3	6.3	NI	NI
		*P. aeruginosa*	1.1 × 10^6^	<1	<1	<1	<1	6.8	6.8	6.8	6.8	6.8	NI	NI
		*E. coli*	8.9 × 10^5^	<1	<1	<1	<1	6.6	6.6	6.6	6.6	6.6	NI	NI
		*C. albicans*	2.2 × 10^5^	—	—	<1	<1	5.6	—	—	5.6	5.6	—	NI
		*A. niger*	1.1 × 10^5^	—	—	<1	<1	5.0	—	—	5.0	5.0	—	NI

71402	0.0125% benzalkonium chloride	*S. aureus*	5.4 × 10^5^	<1	<1	<1	<1	6.3	6.3	6.3	6.3	6.3	NI	NI
		*P. aeruginosa*	1.1 × 10^6^	<1	<1	<1	<1	6.8	6.8	6.8	6.8	6.8	NI	NI
		*E. coli*	8.9 × 10^5^	<1	<1	<1	<1	6.6	6.6	6.6	6.6	6.6	NI	NI
		*C. albicans*	2.2 × 10^5^	—	—	<1	<1	5.6	—	—	5.0	5.0	—	NI
		*A. niger*	1.1 × 10^5^	—	—	<1	<1	5.0	—	—	4.7	4.7	—	NI

71403	0.005% benzalkonium chloride	*S. aureus*	5.4 × 10^5^	<1	<1	<1	<1	6.3	6.3	6.3	6.3	6.3	NI	NI
		*P. aeruginosa*	1.1 × 10^6^	<1	<1	<1	<1	6.8	6.8	6.8	6.5	6.5	-2.1^a^	-1.8^b^
		*E. coli*	8.9 × 10^5^	<1	<1	<1	<1	6.6	6.6	6.6	6.6	6.6	NI	NI
		*C. albicans*	2.2 × 10^5^	—	—	<1	<1	5.6	—	—	1.7	1.7	—	NI
		*A. niger*	1.1 × 10^5^	—	—	<1	<1	5.0	—	—	5.0	5.0	—	NI

NI: no increase means that the increase in test strain did not exceed 0.5 log from the previous measurement time; ^a^ indicates that the increase in test strain was 2.1 log, when compared with that on days 7 and 28; ^b^ denotes that result of day 28 is comparable with that of day 14, and the increase in the number of test strain was 1.8 log.

## Data Availability

The data used to support the findings of this study are available from the corresponding author upon request.
